# Optical Manipulation of nanoparticles by simultaneous electric and magnetic field enhancement within diabolo nanoantenna

**DOI:** 10.1038/s41598-017-13201-w

**Published:** 2017-10-09

**Authors:** Nyha Hameed, Ali Nouho Ali, Fadi I. Baida

**Affiliations:** 1 Département d’Optique P.M. Duffieux, Institut FEMTO-ST, UMR 6174 CNRS, Université Bourgogne Franche–Comté, 15B Avenue des Montboucons, 25030 Besançon Cedex, France; 2 Al Muthanna University, College of Science, Department of Physics, Al Muthanna, Iraq

## Abstract

In this paper, we propose and numerically simulate a novel optical trapping process based on the enhancement and the confinement of both magnetic and electric near-fields by using gold Diabolo Antenna (*DA*). The later was recently proposed to generate huge magnetic near-field when illuminated by linearly polarized wave along its axis. Numerical 3*D* – *FDTD* simulation results demonstrate the high confinement of the electromagnetic field in the vicinity of the *DA*. This enhancement is then exploited for the trapping of nano-particles (*NP*) as small as 30 nm radius. Results show that the trapping process greatly depends on the particle dimensions and that three different regimes of, trapping at contact, trapping without contact, or pushing can be achieved within the same *DA*. This doubly resonant structure opens the way to the design of a novel generation of efficient optical nano-tweezers that allow manipulation of nano-particles by simply changing the operation wavelength.

## Introduction

Optical trapping occurs when the radiation pressure exerted on a particle by an electromagnetic wave is compensated by the gradient forces. The latter are directly linked to the electromagnetic-field spatial distribution around and inside the particle. Thus, highly focused Gaussian beams are firstly used to demonstrate 2D trapping in the transversal direction (perpendicular to the beam propagation direction) due to the presence of electric field gradient along this direction. Unfortunately, 3D trapping with such beams is a very hard task due to the radiation pressure in the longitudinal direction that tends to push the particles along the light propagation direction. The combination of, at least, two propagating beams^1^ or high efficient beam shaping process^2,3^ is needed to get longitudinal confinement^4–6^ in view of achieving 3D trapping. Nano-antennas (*NA*) are ideal candidates to confine light down to the nano-scale and thus to generate high gradient electromagnetic field^7–13^ that can be exploited to trap very small particles. More precisely, monopole, dimmer, bowtie-shaped or bowtie nano-aperture antennas have been recently proposed and used to achieve efficient optical trapping of nano-particles as small as 20 nm-radius. In 2011, a review paper by Juan *et al*.^14^ discusses several of such plasmonic structures in the context of optical trapping. In all these cases, the electric field confinement, induced by the nano-antenna resonance, is at the origin of the force enhancement and non-resonant particles are usually considered to insure a weak coupling with the *NA* and thus a small modification of the resonant mode distribution. The case of metallic or high-index particles is very interesting since they can exhibit proper resonances (electric and/or magnetic dipolar or multipolar). Their coupling with a resonant *NA* will lead to very interesting and rich effects that can be exploited to control the optical force^15,16^.

## Proposed geometry and radiation pressure

**Figure 1:**
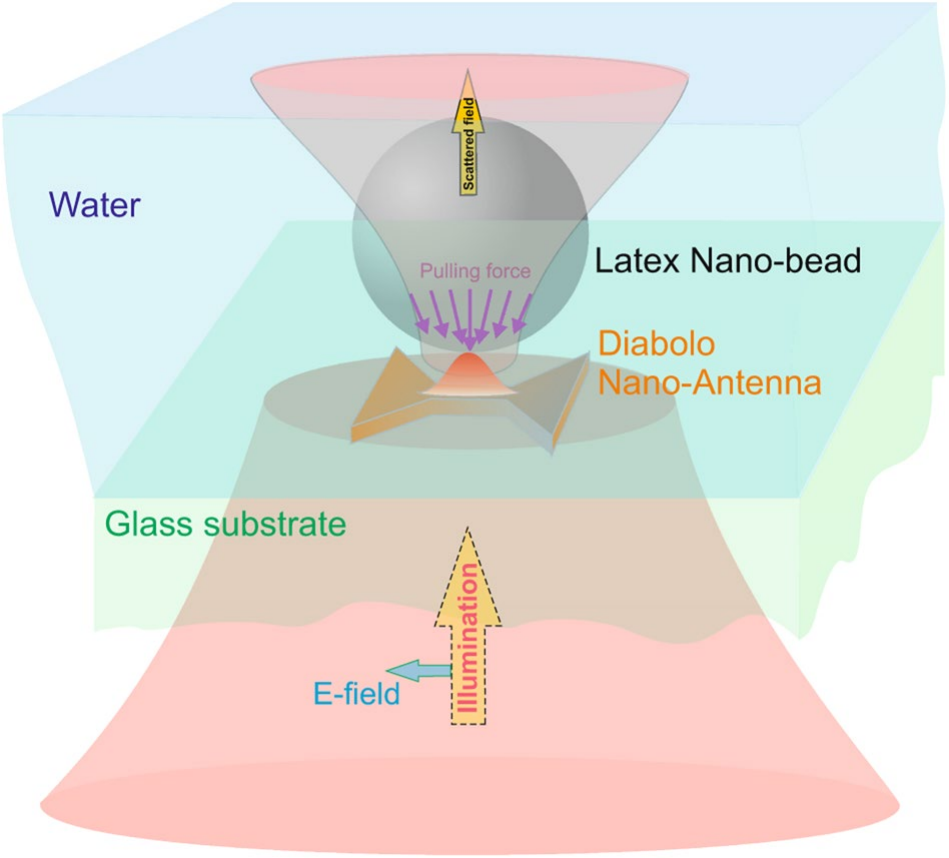
Schematic of the proposed optical nano-tweezers based on Diabolo nano Antenna (*DA*) deposited on a glass substrate and illuminated by a linear polarized beam along the *DA* axis direction. The nanoparticle to be trapped is supposed to be emerged in a liquid on the top side of the glass substrate.

**Figure 2:**
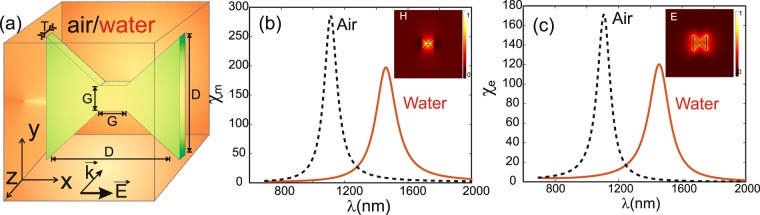
(**a**) Schematic of the diabolo nano-antenna. *D* is the total length in both x and y directions, *G* is the junction length and *T* is the thickness in z direction. (**b**) Magnetic and (**c**) electric enhancement factors of a diabolo antenna made in gold with *D* = 135 nm, *G* = 25 and *T* = 20 nm immersed in air (black dashed lines) and in water (red solid lines). Insets of (**b**,**c**) show respectively the magnetic and electric field distributions at resonance (in air at ${\lambda }_{res}^{air}=1112$ nm) along a transverse (*xOy*) plane at 5 nm above the *DA*.

In this paper, we propose a novel kind of efficient optical tweezers where forces are issued from the increasing and confining of both the electric and the magnetic fields. The proposed structure (see Fig. 1) is based on a gold diabolo nano-antenna (*DA*)^17^ that allows the emergence of a high induced magnetic dipole (in the central zone) in contrast with gap-based nano-antennas where only electric resonance can take place. The origin of the magnetic dipole is linked to a charge transfer between the two metallic arms of the *DA* through its thin central part^18^ which allows controlling of the resonance wavelength by changing the central metal junction dimension. When deposited on a glass substrate and illuminated perpendicularly to its axis (see Fig. 1), the *DA* generates electromagnetic field gradient at resonance that can be exploited to faithfully trap nano-particles. The proposed geometry is given in Fig. 2a. It results from extensive Finite Difference Time Domain (FDTD^19^)-based numerical simulations (homemade code) that were conducted in order to optimize (see the Methods section) the enhancement of both magnetic and electric near-fields^11^. The obtained geometry exhibits a total length of *D* = 135 nm, a junction length and a thickness of *G* = 25 nm and *T* = 20 nm respectively. The dispersion properties of the metal are taken into account in the FDTD simulations as described in the Methods section. The magnetic *χ*_*m*_ and electric *χ*_*e*_ enhancement factors (ratio of the square modulus of the field with *DA* to the same quantity without the *DA*) at 5 nm above the *DA* center (from the transmission side) are shown in Fig. 2b,c for two cases of a *DA* immersed in air or in water. The latter case is more appropriate in common optical trapping experiments due to the particle weight compensation by the buoyancy.

In Fig. 2b,c, the enhancement factors correspond to an incident polarization directed along the *DA* axis. As expected, both fields are efficiently enhanced over a broadband spectral range (resonance with small quality factor ~10). Indeed, one obtains ${\chi }_{e}^{air}=171.7$ and ${\chi }_{e}^{{H}_{2}O}=120.3$ at ${\lambda }_{res}^{air}=1112$ nm and ${\lambda }_{res}^{{H}_{2}O}=1458$ nm respectively. Note that the resonance wavelength (*λ*_*res*_) evolves almost proportionally to the refractive index of the medium surrounding the *DA* $({\lambda }_{res}^{{H}_{2}O}\times {n}_{air}\simeq {\lambda }_{res}^{air}\times {n}_{{H}_{2}O})$. Moreover, the magnetic field responses presented in Fig. 2b show larger enhancement factors than for the electric field with ${\chi }_{m}^{air}\simeq 285.8$ and ${\chi }_{m}^{{H}_{2}O}\simeq 197.2$ at the resonance wavelengths. These large enhancements could lead to increase the optical forces exerted on particles placed in front of the *DA* according to the expression of the Maxwell stress tensor that involves both the magnetic and electric fields:

1 $$\vec{F}=\mathop{\int \int }\limits_{s}\overleftrightarrow{T}\cdot \vec{n}\,ds-{\varepsilon }_{r}{\varepsilon }_{0}\frac{{\rm{\partial }}}{{\rm{\partial }}t}\,[\int \,\int \,{\int }_{V}\,(\vec{E}\times \vec{H})\,d\tau ]$$

where, $\overleftrightarrow{T}$ is the Maxwell stress tensor with elements defined by:

2 $${\overleftrightarrow{T}}_{ij}={\varepsilon }_{r}{\varepsilon }_{0}({E}_{i}\cdot {E}_{j}-{\textstyle \tfrac{1}{2}}{\delta }_{ij}{\vec{E}}^{2})+{\mu }_{0}({H}_{i}\cdot {H}_{j}-{\textstyle \tfrac{1}{2}}{\delta }_{ij}{\vec{H}}^{2})$$

$\vec{E}$ and $\vec{H}$ are the electric and magnetic fields respectively. *i* and *j* are subscripts that take the value *x*, *y* or *z* and *δ* is the Kronecker delta function. *ε*_0_, *ε*_*r*_ and *μ*_0_ are the vacuum permittivity, the relative permittivity of the host medium and vacuum permeability respectively. The enhancement of both $\vec{E}$ and $\vec{H}$ will contribute to increase the resulting optical force. Unfortunately, the insets of the Fig. 2b,c show that, in contrast with the magnetic field, the electric field enhancement mainly occurs at the *DA* corners and not at its center. As it will be demonstrated in the following, this discrepancy between the spatial location of the two field confinements confers to the *DA* a very interesting character that allows it to trap specific nano-particles (according to their dimensions) in a non-contact regime (at distance from the *DA*) despite that the confinement is more intense at the *DA* center.

**Figure 3:**
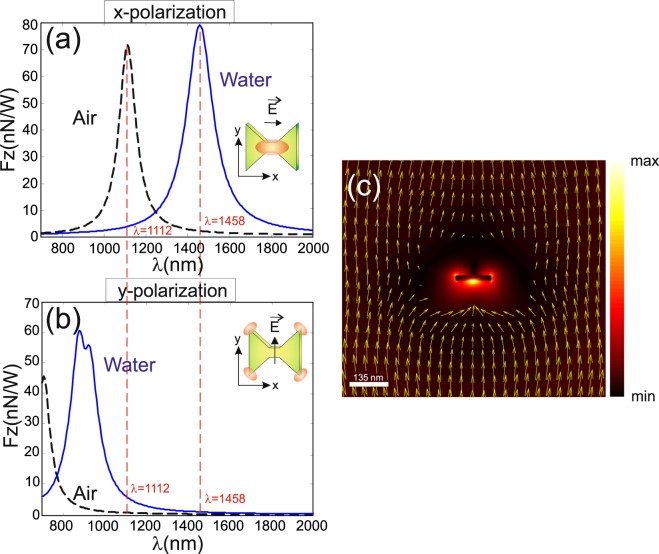
Calculated longitudinal forces exerted by an *x*–polarized (**a**) and *y*–polarized (**b**) plane wave illuminating the *DA* at normal incidence. (**c**) Shows the energy flow distribution (in color map) passing through the middle of the *DA* in a vertical *xOz* plane in the case of *x*–polarization. Yellow arrows correspond to the tangential Poynting vector component. The geometrical parameter of the *DA* are the same as in Fig. 2.

**Figure 4:**
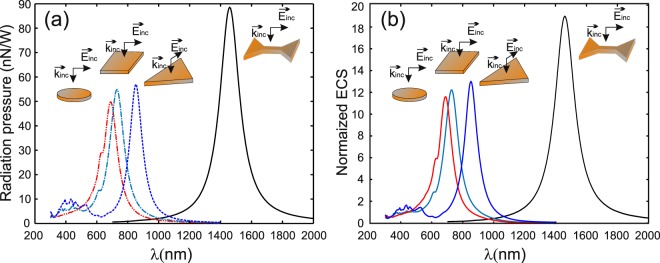
(**a**) Radiation pressure spectra of the three geometries studied in ref.^20^ in addition to the *DA* when illuminated at normal incidence (perpendicular to their average plane) by a linear polarized plane wave. (**b**) Corresponding normalized extinction cross sections (ECS). Note that the four *NP*s has the same volume *V* = 2 · 10^−22^ *m*^3^ and thickness *T* = 20 nm and the triangle is equilateral.

First, we have calculated the optical force generated on the *DA* itself when illuminated by a plane wave linearly polarized along its axis (*x*–axis here to induce *DA* resonance) and along the perpendicular direction (*y*–polarized wave). Indeed, the redistribution of the electromagnetic field around the *DA* due to its resonance can lead to enhance the radiation pressure. This is verified through the results presented in Fig. 3a,b where longitudinal (along the illumination direction) resulting force, per unit of incident light power on the *DA*, is shown for the two cases as a function of the wavelength. A maximum force appears when the *DA* resonates and its value is almost 122 times greater than the off-resonance case for a *DA* immersed in air (@*λ* = 1112 nm) and 92 times larger in water (@*λ* = 1458 nm). The same phenomenon may exist for other particle geometries providing electromagnetic (electric and/or magnetic) resonances^20,21^. In the present case of a resonant *DA*, a light funneling effect takes place and it contributes to enhance the radiation pressure on the *DA* and pushes it along the light propagation direction. This funneling effect is shown on Fig. 3c where yellow arrows indicate the tangential Poynting vector component in a vertical plane (*xOz*) passing through the *DA* center. Nevertheless, we have verified that the radiation pressure generated by the *DA* resonance is as efficient as in the case of the three geometries studied in ref.^20^ as shown on Fig. 4a. In all four cases, same metal volume and thickness are considered (*V* = 2 · 10^−22^ *m*^3^ and *T* = 20 nm). On the other hand, generally, the radiation pressure can be linked to the extinction cross section (*ECS*) of each particle: the more the *ECS* of the particle is, the more the modification of the surrounding electromagnetic field will be, and the more the gradient force will be either. Figure 4b shows the normalized *ECS* calculated by Total Field Scattered Field (TFSF)-FDTD technique for the four geometries when illuminated by a linearly polarized plane wave. Unfortunately, even if at resonance the *DA* behaves as a magnetic dipole, there is a small electric contribution (probably quadripolar contribution) due to the electric field enhancement occurring at the *DA* corners as shown on the inset of Fig. 2c. The relationship between the radiation pressure and the *ECS* is then no more explicit as in the case of pure dipolar resonance and would involve both electric and magnetic polarizabilities of the *NP*s^22^. At first glance, assuming pure magnetic (electric) dipolar resonance of the *DA* (triangle), the magnetic polarizability of the *DA* seems to recover the value of the electric polarizability of the triangle because the *F*_*z*_/*ECS* ratio is almost the same (4.5 × 10^−9^) for the two *NP*s at their respective resonance. Further investigations, such as the study of the coupling between these particles with another resonant system (photonic crystal for example^23^), can be done to determine the electric and magnetic contributions to the resonance properties of such *NP*s.

## Force results: the three different regimes

**Figure 5:**
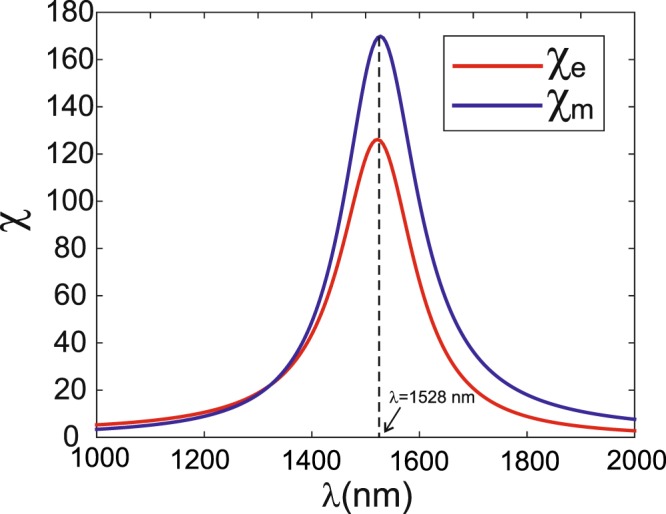
Electric (red curve) and magnetic (blue curve) enhancement factors in the case of a *DA* in water deposited on a glass substrate.

Let us now consider a configuration where the *DA* is used as optical nano-tweezers. It is supposed to be illuminated by a linearly polarized plane wave impinging from a glass substrate on which it is deposited. As commonly used, the superstrate medium, which contains the particles to be trapped, must be a liquid (for instance water with *n* = 1.315) in order to compensate their weight by the Archimedes’ buoyancy. First, we calculated the near-field electric and magnetic enhancement factors as in Fig. 2 measured at 5 nm above the *DA* center. Results are plotted in Fig. 5 and show that the resonance properties (both resonance wavelength and enhancement factors) of the *DA* are affected by the presence of the substrate. This phenomenon was already pointed out for another kind of nano-antennas, such as a bowtie nano-antenna^11^ or the bowtie nano-aperture antenna (*BNA*) for which the resonance properties were examined as a function of the antenna-to-substrate distance^24,25^. In the latter cases, a shift as large as Δ*λ* = 500 *nm* of the resonance wavelength of a *BNA* occurs when the latter moves up over 10 *nm* from the substrate interface. In the present case, the shift is smaller (only Δ*λ* = 70 *nm*) due to the small contrast of the optical index between the substrate (*n* = 1.49) and the superstrate (*n* = 1.315) compared to the case of InP (*n* = 3.17) and air (*n* = 1) considered in the study of ref.^25^. Nevertheless, we should take into account this shift especially if we aim to operate at a given value of the wavelength.

At first glance and compared to the enhancement of the electric field generated at the vicinity of a *BNA*^13^, the *DA* seems to be more efficient to act as a nano-tweezers. Unfortunately, there is a major discrepancy between the two nano-antennas: contrarily to the *BNA* where the optical force is due to the light passing through it, a predominant part of the force generated on a *NP* placed in front of the *DA* consists on a radiation pressure. This is particulary valid when the *NP* is far from the nano-antenna and/or when it exhibits a large radius. Thus, stable and efficient trapping is only expected for small *DA*-to-*NP* distances and small *NP*s. Nevertheless, the effect of this background illumination may lead, according to the *NP* dimension, to a trapping position without contact (compensation of the radiation pressure by the gradient force). To investigate all these assumptions, we have made extensive simulations to quantify the force exerted on dielectric *NP*s. We present here three different studies where only the *NP* position along the vertical axis passing by the *DA* center (perpendicular to the substrate plane) is considered. The calculated vertical force *F*_*z*_ (the only non zero component of the force) is normalized by the total energy impinging the *DA*. In all simulations, the dielectric permittivity of the *NP* is modeled with a subgriding technique in order to accurately describe its spherical geometry.

**Figure 6:**
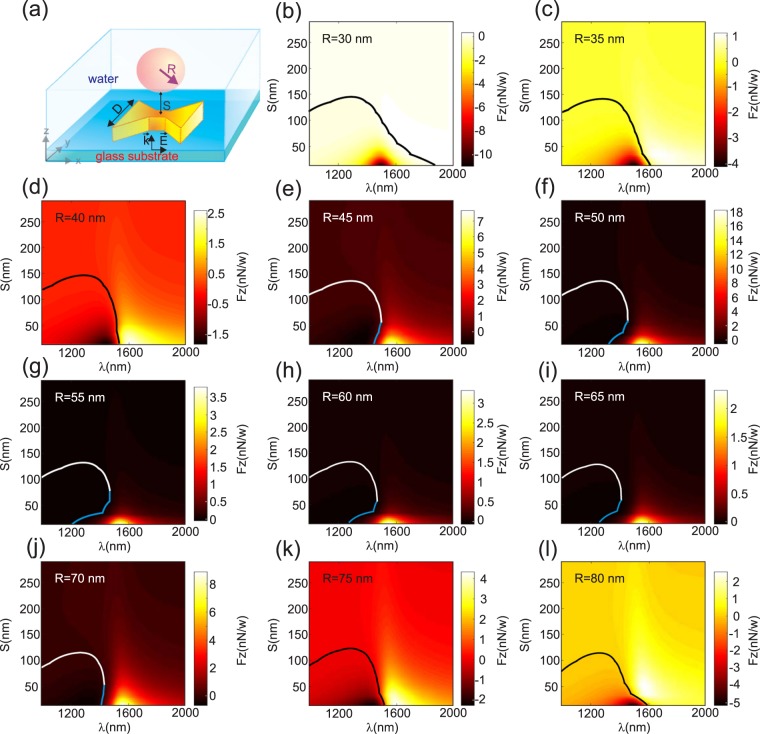
(**a**) Schematic of the simulated configuration giving all the geometrical parameters: the *DA* has the same geometry as in Fig. 2. (**b**–**l**) Numerical results of the vertical force (*F*_*z*_) spectrum as a function of the *DA*-to-*NP* distance (*S*) for different *NP* radius varying from *R* = 30 nm to *R* = 80 nm per 5 nm step. The solid lines correspond to *F*_*z*_ = 0 N/W delimiting repulsive regime from attractive one. Blue part of this line appearing in (**g**–**j**) indicates a stable regime of trapping without contact.

**Figure 7:**
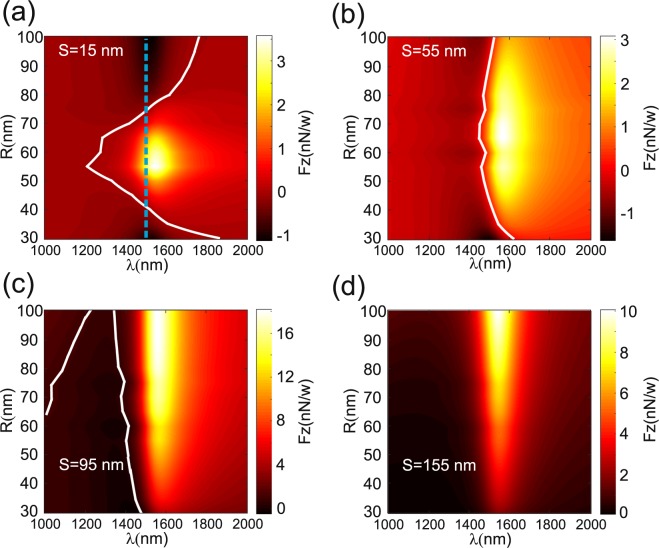
Numerical results of the vertical force (*F*_*z*_) spectrum as a function of the *NP* radius (*R*) for different *DA*-to-*NP* distance (*S*): (**a**) *S* = 15 nm, (**b**) *S* = 55 nm, (**c**) *S* = 95 nm and (**d**) *S* = 155 *nm* . The vertical dashed blue line in (**a**) corresponds to the resonance wavelength of the *DA*. The white lines correspond to *F*_*z*_ = 0 *N*/*W*.

In the first study, the radius of the *NP* is fixed while its position varies together with the illumination wavelength. In the second study, the distance *S* is fixed and the two other parameters vary (*R* and *λ*). Last study is done when fixing the wavelength and varying *R* and *S*. The corresponding results are shown on Figs 6, 7 and 9 respectively. For each study, attractive and repulsive zones are indicated on the corresponding figure together with the colored separation line (*F*_*z*_ = 0) that corresponds to the optical trapping of the *NP*. According to the coordinate system in Fig. 1, a negative value of *F*_*z*_ corresponds to an attractive force while positive one leads to push the *NP* away from the *DA*.

In Fig. 6a, we recall the geometric parameters of the studied configuration and we depict the 11 force spectra for values of the *NP* radius varying from *R* = 30 nm to *R* = 80 nm by 5 nm step. The solid line on all the sub-figures corresponds to *F*_*z*_ = 0 i.e. a possible trapping position far from the *DA* (trapping at distance). For *R* = 30; 35; 75 and 80 nm (see Fig. 6b,c,k,l) attractive force occurs at small distances *S* < 140 nm and at wavelength values ranging from *λ* = *λ*_*res*_ = 1528 nm to *λ* = 1800 nm. One notes that when trapping occurs, it corresponds necessarily to a contact between the *DA* and the *NP*. The white solid lines correspond then to an unstable trapping characterized by a maximum of the potential instead of a minimum (potential well).

Nevertheless, for radius value from *R* = 40 nm to 70 nm, the attractive zone becomes limited to smaller wavelength values than *λ*_*res*_ = 1528 nm. In fact, as shown on Fig. 6d, *F*_*z*_ vanishes in the vicinity of the resonance. This is probably due to the fact that the radiation pressure becomes larger while the gradient force is almost kept the same. According to the insets of the Fig. 2b,c, the force is mainly due to the the magnetic field confinement that occurs at the *DA* center. We have verified that the contribution of the magnetic field to the force is generally of the order of magnitude of the electric field one. Nevertheless, these two contributions have an opposite sign and thus can compensate each other. For $R\in [45;70]\,{\rm{nm}}$ (see Fig. 6e–j), a very interesting phenomenon appears where the attractive zone is preceded by a repulsive one when *S* increases. This corresponds to a stable trapping without contact between *NA* and *NP* as depicted by the blue lines in Fig. 6g,h.

We have verified that this trapping at distance is only obtained for *NP*s with $R\in [45;70]\,{\rm{n}}{\rm{m}}$. Nonetheless, the wavelength intervals, where this regime occurs, vary with the radius value as it will be shown in the following. When the *NP* radius increases (see Fig. 6k,l), the attractive zone shifts toward larger values of the wavelength due to the efficient overlap between the *NP* and the electric field of the *DA* generated at its corners. By the way, another value of *R* exists for which the vertical force vanishes at resonance (here *R* = 75 nm as seen on Fig. 6k).

In Fig. 7, four values of the *DA*-to-*NP* distance are considered: *S* = 15; 55; 95 and 155 nm. For *S* = 15 nm, Fig. 7a shows that attractive force occurs at small distance *S* for all *NP* radius and small values of the wavelength ($\lambda \in [1000;1800]\,nm$). The attractive and repulsive zones are separated by white lines. The dashed blue line on the figure corresponds to the *DA* resonance wavelength. Along this line, when we increase the distance *S* from 55 nm to 95 nm, the attractive zone becomes smaller and it is globally blue-shifted as shown on Fig. 7b,c. The maximum of the repulsive force always appears nearby the resonance wavelength due to the funnel effect (see Fig. 3c) induced by the *DA* leading to increase the radiation pressure on the *NP*. For larger distance values *S* (>155 nm), the attractive zone almost vanishes in the considered wavelength range and the *NP* is pushed away from the *DA* as shown in Fig. 7d. This phenomena, as expected, occurs as a result of the background illumination that becomes predominant when the *NP* is far from the *DA*.

**Figure 8:**
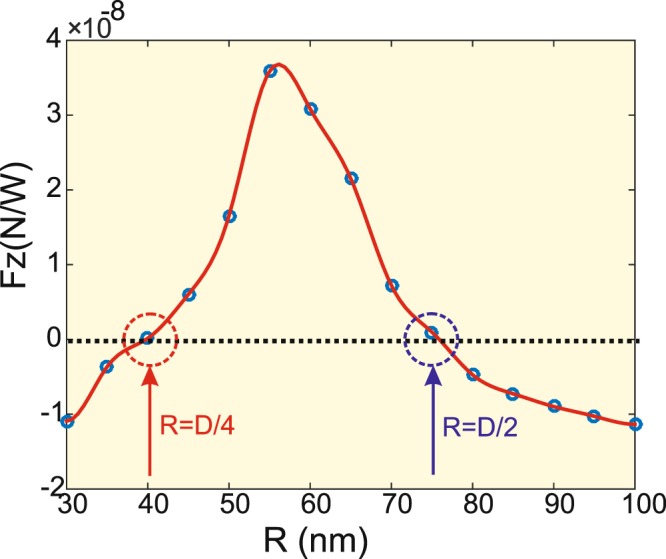
Numerical results of the vertical force as a function of the *NP* radius *R* for *DA*-to-*NP* distance *S* = 15 nm. The illumination wavelength corresponds to the *DA* resonance *λ*_*res*_ = 1528 nm. The vertical arrows indicates a stable trapping without contact.

To get more physical insight on the *DA*-*NP* interaction at the resonance wavelength, we plot on Fig. 8 a cross-section made on the result of Fig. 7a at *λ*_*res*_ (vertical dashed blue line). This plot gives the optical force exerted on the *NP* as a function of its radius (*R*) when it is placed in front of a resonant *DA* (*λ* = *λ*_*res*_ = 1528 nm) and at a fixed distance *S* = 15 nm. This allows direct determination of the *NP* radii (here two values) for which trapping occurs (zero vertical force) at this specific distance. The first value (see red vertical arrow on Fig. 8) is *R* = 40 nm and it almost corresponds to quarter of the *DA* length. For this *NP* dimension, only the field confinement that occurs at the *DA* center is felt by the *NP* and seems to compensate the radiation pressure exerted on the *NP* parts that are out of the *DA* shadow. The second value (see the blue vertical arrow on Fig. 8) is *R* = 70 nm corresponding to a *NP* size that is almost equal to the *DA* one (*D* = 135 nm). In this case, the trapping is obtained thanks to the effect of the electric field confinement at the *DA* corner that acts together with the field confinement at the *DA* center to compensate the radiation pressure growth.

**Figure 9:**
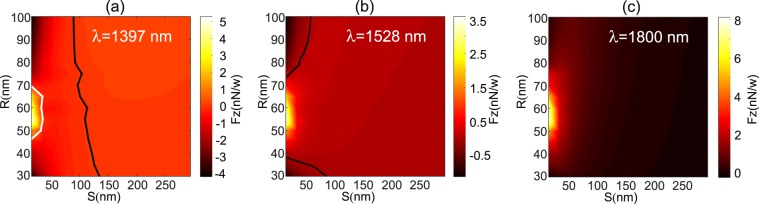
Numerical results of the vertical force (*F*_*z*_) as a function of *NP* radius (*R*) and *DA*-to-*NP* distance (*S*) for different operation wavelengths: (**a**) *λ* = 1397 nm, (**b**) *λ* = *λ*_*res*_ = 1528 nm and (**c**) *λ* = 1800 nm. The black lines correspond to an unstable trapping while the white line in (**a**) indicates stable trapping without contact. In (**c**) the vertical force is always positive and never vanishes.

Experimentally, it is more adequate to operate at a fixed wavelength value. Figure 9 shows the variations of the vertical force for three different values of the wavelength when both *R* and *S* vary. Figure 9a corresponds to a wavelength smaller than the resonance one. In this case, a stable trapping at distance (white solid line) may occur for $R\in [45;70]\,{\rm{nm}}$ providing initial *DA*-to-*NP* distance smaller than 100 nm. This can be obtained by increasing the concentration of *NP*s in the liquid. The black line corresponds to an unstable trapping as mentioned above. At the resonance wavelength, the repulsive zone spreads over almost the total window and two small attractive areas remain for *R* > 70 nm and *R* < 40 nm only if *S* < 90 nm. At this peculiar wavelength, only trapping with contact may occur and *NP*s such $R\in [40;70]\,{\rm{n}}{\rm{m}}$ are never trapped as seen on Fig. 9b. This configuration can be exploited to make a *NP* sorting with respect to their dimensions. For larger wavelength value (here *λ* = 1800 *nm*), only repulsive zone exists due to the absence of any light confinement. The radiation pressure is then predominant and only pushing force acts on the *NP* as shown in Fig. 9c.

**Figure 10:**
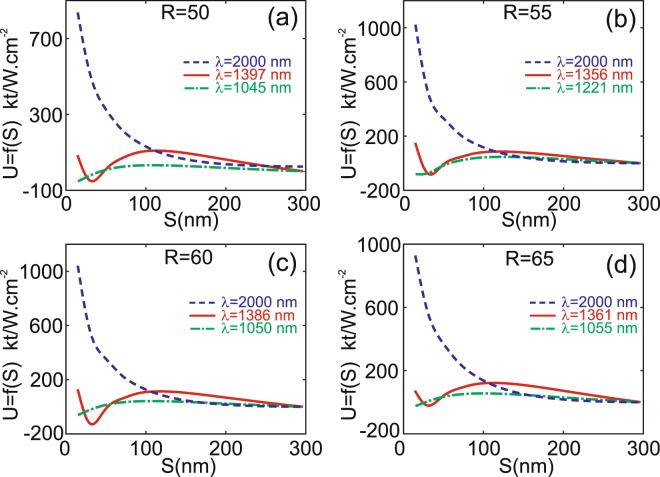
Potential energy for different *NP*s radii: *R* = 50 nm in (**a**), *R* = 55 nm in (**b**), *R* = 60 nm in (**c**) and *R* = 65 nm in (**d**). For each case, three different regimes can be obtained by changing the operation wavelength value. Solid red lines correspond to a trapping at distance (potential well), Dashed dotted green lines to trapping at contact and dashed blue lines to pushing regime.

In order to point out how to manipulate *NP* within a *DA*, we extract three different scenarios that correspond to pushing, trapping at distance or trapping at contact that can exist for a given *NP* (fixed *R* value) only by changing the operation wavelength. We only consider four values of the *NP* radius (*R* = 50; 55; 60 and 65 nm) for which trapping at distance can occur. As shown in Fig. 10 where the potential $(U=-{\int }_{{\rm{\infty }}}^{r}\,\vec{F}(r)\,\vec{d}r)$ is plotted as a function of *S*, one can always find a wavelength value that corresponds to one of the three scenarios. In all cases, the trapping at distance exhibits a potential well larger than 10 *kT* providing an illumination power larger than 5 *mw*. In all figures, the red curves represent the trapping without contact regime where a potential well exists for *S* ≠ 0 while green curves correspond to the case of trapping with contact where the potential well occurs for S < 15 nm. Due to the symmetry of the configuration, there is no lateral components of the force and only the vertical one is non zero along the *z*-axis. On the contrary, the blue curves correspond to wavelength values for which the *NP* is pushed away from the *DA* (no trapping). Finally, a sorting process can be envisaged at this wavelength value where all *NP*s with $R\in [40;70]\,{\rm{n}}{\rm{m}}$ are pushed away from the *DA* and other *NP* radii (<150 nm) are trapped.

## Conclusion

In summary, we have theoretically and numerically studied the optical trapping of polystyrene *NP*s by using Diabolo nanoantenna (*DA*). The latter shows that this configuration is able to confine the electric and the magnetic fields of the incident illumination with large enhancement factor. Numerical simulations were performed to determine the mechanical interaction between the *DA* and the *NP*s. Optical force exerted on the *NP* has been studied as a function of size, wavelength and distance. This study demonstrates the ability of the *DA* to manipulate small *NP*s through different regimes: trapping at contact, trapping at distance or pushing by simply changing the operation wavelength. This was achieved thanks to the presence of both electric and magnetic field confinements occurring at different spatial locations on the *DA*. *NP* manipulation is then made possible by simply changing the operation wavelength. The design of the *DA* can be optimized to fulfill the experimental constraints (sources and detectors) with respect to the *NP* dimensions.

## Methods

All the numerical simulations were done with home-made Finite Difference Time Domain (FDTD) codes developed by FB in Fortan language. These codes include Perfectly Matched Layers as absorbing boundary conditions an a non-uniform meshing in order to faithfully describe the small features of the structure. For example, the *DA* is entirely discretized with small cubes of 5 × 5 × 5 nm^3^ while a coarse meshing (25 × 25 × 25 nm^3^) is applied elsewhere. A gradual transition between the two meshing zones is respected to avoid parasitical reflections related to this spatial step change. The metal dispersion of Gold is modeled through a Drude model given by ${\varepsilon }_{r}(\omega )=1-\tfrac{{\omega }_{p}^{2}}{\omega (\omega +i\gamma )}$ with *ω*_*p*_ = 1.2874 × 10^16^ *rad*/*s* and *γ* = 1.275 × 10^14^ *rad*/*s*. The calculation of the *NP*’s Extinction Cross sections is performed using a Total-Field/Scattered-Field technique that was implemented especially for this purpose. The electromagnetic field components are Fourier transformed during the temporal loop of the FDTD and recorded over the six faces of a cube (or parallelepiped) that encloses the *NP*. The integral of Eq. 1 is done under Matlab environment with codes allowing determination of the force values through the determination of the Maxwell stress tensor given by Eq. 2. In harmonic regime, the second term of Eq. 1 vanishes due to the cross product of $\vec{E}$ by $\vec{H}$ that leads to a time dependence term in *e*^−2*iωt*^ and then to a zero temporal mean value (integral term in Eq. 1). The closed surface (*s*) considered to calculate the force by the flux of the Maxwell stress tensor (see Eq. 1) must surround the *NP* while remaining in the same medium (same value of *ε* and *μ* over all the surface). This is directly linked to the conservation of the energy (Poynting flux) theorem. Consequently, and, due to the spatial discretization used in the *FDTD*, the minimum value of the *DA*-to *NP* distance *S* is 3 times the spatial cell length in order to set the surface (*s*) at one *FDTD* cell from the *NA* and one *FDTD* cell from the *NP*. Near the structure, the cell size is 5 nm leading to *S*_*min*_ = 15 nm. The optimization of the *DA* geometry was done according to some criteria: the first one is the operation wavelength (resonance wavelength) that must be in the NIR region and more precisely between 1450 nm and 1550 nm to be compatible with available sources and detectors in view of experimental tests. In addition, the configuration must exhibit maximum enhancement factor for both magnetic and electric near-fields recorded in the vicinity of the *DA* center. For this purpose, the thickness (T) was varied from 15 nm to 50 nm, the *DA* total length (D) from 100 nm to 170 nm and the junction size (G) from 15 nm to 55 nm. All these parameters were varied by a step of 5 nm. Thus, we ran 128 3D-FDTD simulations (32 in water and 32 in air with and without substrate). Figure curves and maps are also plotted with Matlab and presentations are then enhanced with CorelDRAW. We wish to emphasize that the results presented in this paper were obtained by FDTD simulations which consumed a total CPU-time of more than 3 years.
