# Author Correction: Optical Manipulation of nanoparticles by simultaneous electric and magnetic field enhancement within diabolo nanoantenna

**DOI:** 10.1038/s41598-018-30985-7

**Published:** 2018-09-07

**Authors:** Nyha Hameed, Ali Nouho Ali, Fadi I. Baida

**Affiliations:** 1 Département d’Optique P.M. Duffieux, Institut FEMTO-ST, UMR 6174 CNRS, Université Bourgogne Franche–Comté, 15B Avenue des Montboucons, 25030 Besançon Cedex, France; 2 Al Muthanna University, College of Science, Department of Physics, Al Muthanna, Iraq

Correction to: *Scientific Reports* 10.1038/s41598-017-13201-w, published online 09 October 2017

The original version of this Article omitted an affiliation for Nyha Hameed. The correct affiliations for Nyha Hameed are listed below:

Département d’Optique P.M. Duffieux, Institut FEMTO-ST, UMR 6174 CNRS, Université Bourgogne Franche–Comté, 15B Avenue des Montboucons, 25030, Besançon Cedex, France

Al Muthanna University, College of Science, Department of Physics, Al Muthanna, Iraq

This has now been corrected in the HTML and PDF versions of this Article.

